# Automatic 3D Building Reconstruction from OpenStreetMap and LiDAR Using Convolutional Neural Networks

**DOI:** 10.3390/s23052444

**Published:** 2023-02-22

**Authors:** Marcos Barranquero, Alvaro Olmedo, Josefa Gómez, Abdelhamid Tayebi, Carlos Javier Hellín, Francisco Saez de Adana

**Affiliations:** Computer Science Department, Universidad de Alcalá, 28801 Alcalá de Henares, Spain

**Keywords:** OpenStreetMap, LiDAR, convolutional neural network, 3D reconstruction, transfer learning, machine learning

## Abstract

This paper presents the implementation of an automatic method for the reconstruction of 3D building maps. The core innovation of the proposed method is the supplementation of OpenStreetMap data with LiDAR data to reconstruct 3D urban environments automatically. The only input of the method is the area that needs to be reconstructed, defined by the enclosing points in terms of the latitude and longitude. First, area data are requested in OpenStreetMap format. However, there are certain buildings and geometries that are not fully received in OpenStreetMap files, such as information on roof types or the heights of buildings. To complete the information that is missing in the OpenStreetMap data, LiDAR data are read directly and analyzed using a convolutional neural network. The proposed approach shows that a model can be obtained with only a few samples of roof images from an urban area in Spain, and is capable of inferring roofs in other urban areas of Spain as well as other countries that were not used to train the model. The results allow us to identify a mean of 75.57% for height data and a mean of 38.81% for roof data. The finally inferred data are added to the 3D urban model, resulting in detailed and accurate 3D building maps. This work shows that the neural network is able to detect buildings that are not present in OpenStreetMap for which in LiDAR data are available. In future work, it would be interesting to compare the results of the proposed method with other approaches for generating 3D models from OSM and LiDAR data, such as point cloud segmentation or voxel-based approaches. Another area for future research could be the use of data augmentation techniques to increase the size and robustness of the training dataset.

## 1. Introduction

In recent years there has been an increasing need to generate 3D urban environments that contain detailed data such as roads, buildings, vegetation information, or ground surface information [1]. There are important applications of 3D urban environments in a wide range of fields, from engineering and architecture to public safety. For example, in GPS navigation systems [2], which can be of great help in vehicle and public transport navigation, route planning, and avoiding obstacles such as construction or congested roads. This links into the field of computer vision, where it can be used for object, person, and vehicle detection in security applications such as surveillance of urban areas or detection of suspicious objects [3]. One of the most common uses of 3D urban environments is in simulators. These three-dimensional environments can be used to create realistic simulations of urban scenarios, which can be of great help in driver training, preparing personnel for emergency situations, and simulating evacuation situations in the event of natural disasters [4]. In addition, 3D urban environments can be useful in simulating situations related to forest fire prevention and control, as well as radio propagation in urban areas [5].

Although highly accurate 3D maps are required in many of these applications, it is difficult to create a consistent method that automatically generates these 3D urban environments. Many of the existing approaches are based on a single data source and take full advantage of that data to create the most accurate environments possible; however, the information that allows for reconstructing the 3D environment is not always complete. Obviously, this can limit the precision and quality of the obtained results. Therefore, new approaches are needed that combine different types of data and use advanced image processing and machine learning techniques to automatically create high quality 3D urban environments.

Before defining the hypothesis of this research, we briefly review the current state of the research field.

### 1.1. LiDAR

LiDAR is a system that uses lasers to determine distances. It is used for navigation and mapping, where it can provide precise distance measurements. Recent works have shown that LiDAR can be useful in multiple fields, such as archaeology [6], flooding [7], forest biomass estimation [8], and autonomous vehicle control [9]. In relation to urban environments, satellite imagery or aerial measurements can be used to include information on buildings and non-natural elements of the landscape. The data collected from these measurements can be used to generate a DSM that includes the terrain altitude, geometry, and position of non-natural landscape elements such as buildings in urban environments. In [10], the authors proposed a method for automatic segmentation of LiDAR point cloud data at different height levels for 3D building extraction. The method scans the point cloud data from the highest to the lowest height level and extracts the building points at each height level. The building points are then clustered into different building segments, and trees and non-building points are removed. The method is based on the assumption that the building points are distributed in a cluster-like manner. The method then can extract roof points of the buildings. It achieves high accuracy, particularly on roofs with shapes that are not flat, where it can read the coplanarity of the points. The results show that the method is able to extract building points at different height levels with high accuracy. However, buildings with low heights are poorly detected, and vegetation may be processed as building points in certain cases. A more exhaustive study was carried out in [11], where an automatic building segmentation method was presented. The authors collected LiDAR data using an unmanned aerial vehicle and used the data to directly segment buildings. They used a dynamic convolutional neural network (DCNN) algorithm to separate buildings and vegetation, then used Euclidean clustering to segment each building. The authors concluded that this method is an effective way to segment buildings using LiDAR data.

LiDAR allows the creation of accurate maps of 3D scenarios, including indoor environments, for applications such as building mapping, autonomous indoor navigation, and obstacle detection in indoor environments. In [12], the authors proposed an improved approach for energy modeling of already-existing buildings. Their solution enables rapid generation of the building geometry from point clouds, increasing the speed with which 3D geometry of existing industrial buildings can be created, and has potential applications in building energy modeling and subsequent thermal simulation. Although 3D building reconstruction was carried out, this algorithm is not automated.

### 1.2. Convolutional Neural Networks

Neural networks can be used to extract relevant features from LiDAR data and use it to classify different types of objects in the scene, such as buildings, vegetation, and other objects. In addition, neural networks can be used to perform segmentation tasks to separate individual buildings from a LiDAR scene. Convolutional Neural Networks (CNN) are used in deep learning to classify images. They can process data from satellite imagery or aerial scans and use information from the curves and shapes of the buildings to classify them into different categories. GIS data such as building location and size information can be used to improve classification accuracy.

In the field of GIS data, several authors and researchers have used neural networks to detect different geospatial datasets. In [13], terrain classification was achieved using a hierarchical deep learning framework with a CNN and a prediction preprocessing step. In [14], the authors used CNN to classify satellite images, comparing various CNN architectures. Even more accurate object detection was achieved in [15] using a multi-scale CNN able to detect objects such as airplanes or ships from high-resolution satellite images. Different neural networks have been used as well; [16] used a generative adversarial network (GAN) and CNN to classify partial discharge patterns in GIS. In [17], the authors used a CNN to classify COVID-19 infection transmission using GIS data and COVID-19 data materials. The authors of [18] used the YOLOv2 open-source deep learning model and GIS-based algorithms to track vehicles, achieving 91% accuracy. In [19], a deep learning model was used to detect cracks in asphalt surfaces, providing surface images for subsequent training and model validation.

An interesting use case of CNN models is to read and transform 2D images to 3D environments. This idea has been applied in works in different fields. In [20], the authors presented a method for reconstructing 3D models of objects from 2D correspondences of points in a single image. The method uses a deformable shape model in which the shape of an object is represented as a linear combination of base shapes. The authors developed an efficient algorithm to solve the proposed convex program, and extended the model to handle outliers in the input 2D correspondences. They applied their method to images of people in different positions or performing actions such as walking, running, jumping, etc., achieving efficient results in terms of accuracy and speed. In [21], the authors show how geometric images can be used to classify and retrieve 3D shapes in 3D shape datasets and propose a CNN architecture for this purpose. Their experimental results showed that this method is capable of classifying and retrieving 3D shapes accurately and efficiently.

### 1.3. OpenStreetMap

OSM is a collaborative project to create a free and editable map of the world. Founded in 2004, it is made up of volunteers who collect and contribute data about roads, buildings, and other features in their local area. The resulting map is free to use and download, and provides an alternative to traditional proprietary maps.

**Figure 1:**
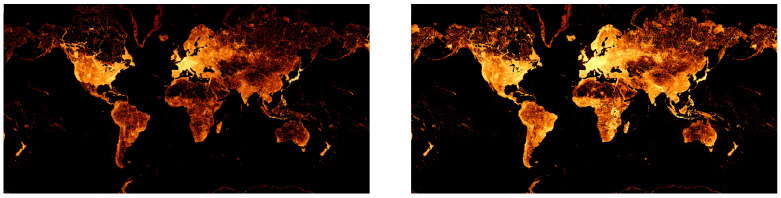
Evolution of the available data provided by OSM. **Left**: Node density map in 2014. **Right**: Node density map in 2022.

A study on the activity of its members has shown that approximately 1.2 million nodes and 130,000 paths are added daily [22]. Especially in metropolitan regions, a large amount of building data has been added recently. Figure 1 shows a comparison between the node density maps for 2014 and 2022 [23]. Here, a lighter color means a higher node density, whereas darker color means a lower node density.

OSM stores a wide variety of information about the features that appear on the map, including roads, buildings, natural features, and more. Among the specific types of information related to buildings that OSM stores are the type of building, its height and shape, its roof height and shape, and more.

OSM has been used in multiple projects and scopes. In [24], the authors provided a framework that analyzes information provided in OSM with the goal of detecting faulty information. OSM has been used in smart cities [25] and in studies of the impact of natural disasters on the built environment [26]. Studies have been conducted to analyze the impact of location-based games on OSM [27]. OSM stores points of interest [28], and has been used in studies comparing inequalities in accessibility [29]. It has been used to analyze the impact of urbanization on green infrastructure [30] and to identify locations along railway networks with the highest tree fall hazard [31]. OSM data can be used to extract the road network of cities [32] or even to extract more complicated roads such as desert roads [33]. In addition, OSM has been used along with other data sources to generate 3D maps of the built environment [34].

In [35], the authors proposed a supervised learning-based approach that classifies buildings into residential or non-residential for different areas of the United States. The model uses existing OSM data that captures non-spatial attributes as well as geometric and topological properties of building footprints, including type of adjacent roads, proximity to parking lots, and building size. The model uses a decision tree and is trained and tested using OSM data on the residence type. The paper shows that this approach achieves accuracy in predicting residence type for selected areas. However, there are more sophisticated methods to infer information than decision trees, such as deep learning, which is the method used in our work.

### 1.4. Hypothesis

According to the state of the art, LiDAR data can be used to improve the accuracy of OSM maps. In this sense, the present paper proposes a method that combines OSM data with LiDAR data to generate more accurate 3D environments. The method proposes using OSM data to create 3D models of buildings and other structures, then using LiDAR data to fill in potential gaps and provide a more detailed view by applying image processing techniques. This can improve the overall accuracy and detail of the map, especially in urban areas where buildings and other structures are densely packed. Additionally, neural networks can be used to process LiDAR data and read certain details of buildings, such as the shape and pitch of roofs. Therefore, we propose a neural network trained on a LiDAR dataset to develop a model capable of automatically extracting information about rooftops. This can improve the accuracy and detail of the building information provided by OSM. For training the neural network, we proposed using LiDAR images of a particular area and extrapolating them to other urban areas with the generated model in order to analyze new images and extract features that can provide more complete OSM data on these new urban areas.

In general, and unlike the works reviewed above, the proposed methodology allows us to answer the following research questions:

1. Whether it is possible to combine OSM data with LiDAR data;
2. Whether it is possible to improve the accuracy of 3D urban environments generated with OSM data through LiDAR data analysis;
3. Whether it is possible to extract features from LiDAR data in order to generate complete OSM data via LiDAR image analysis by creating models based on a convolutional neural network;
4. Whether a model based on a convolutional neural network trained with LiDAR data on a particular region can then extrapolate from those data to analyze new regions and complete the OSM data on these new regions.

The rest of this paper is organized as follows: Section 2 presents the method for generating 3D models of buildings and other structures from OSM data and the process of adding inferred heights and roofs from LiDAR; Section 3 presents the results; finally, Section 4 discusses the limitations of this work, lays out possible future lines of work, and presents our conclusions.

## 2. Materials and Methods

### 2.1. Generating 3D Models of Buildings and Inferring Heights

**Figure 2:**
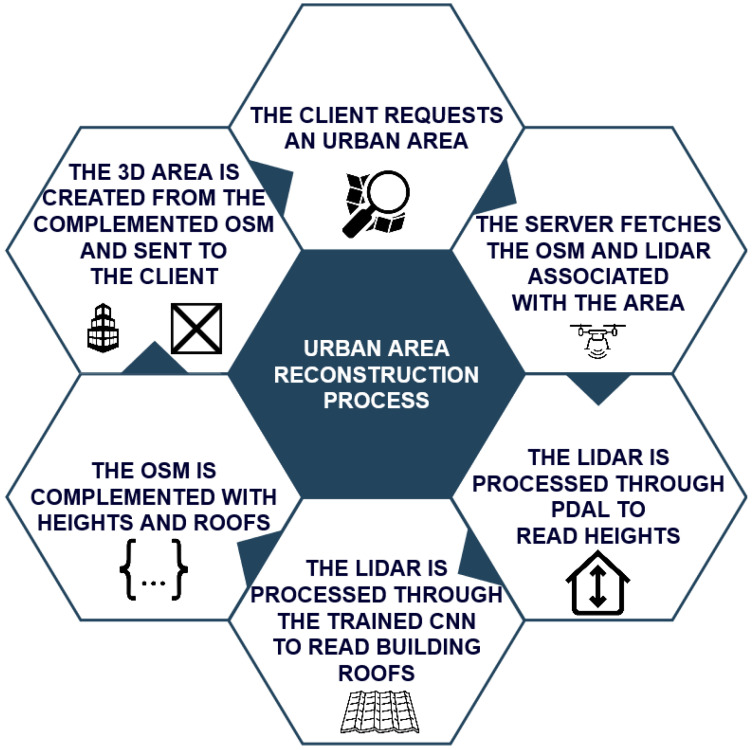
Diagram of the full process used to generate the 3D environment.

To process LiDAR data, we relied on two approaches. The first was to complement the missing building height data from OSM directly with the LiDAR data. For this, a plane segmentation algorithm was used to detect the buildings from the LiDAR data. With the building coordinates available, the floor and roof heights could then be read for each building. The second approach was to complement the roof type data by applying a neural network to identify the roof type of each building. For this, a CNN was trained to identify the roofs of the buildings. LiDAR data were converted to an 8-bit TIFF, then the TIFF file was used as input to the CNN. The CNN model supports a TIF image generated from LiDAR and approximates the roof type to the OSM roof classification, thereby complementing the data. This process can be seen in the diagram in Figure 2.

The developed application used a NodeJS Typescript architecture. The OSM data were fetched from different sources and then transformed to a 3D file containing the urban environment. This process is described in detail below.

Hypertext Transfer Protocol Secure (HTTPS) was used to obtain information about specific map tiles using X, Y, and Z indexes. The obtained information was encoded in JSON, and included detailed descriptions of building geometry within the specified tile, including shape, size, and location.

Tools such as Overpass and OSMBuildings can be used to fetch OSM data. Overpass is a web service for querying the OSM database, while OSMBuildings is a tool for retrieving OSM data as tiles for visualization. Both tools were utilized in the development of the application through an asynchronous process. The data obtained were transformed into GeoJSON, a widely-used open standard format for encoding geographic data structures. GeoJSON data can be easily processed and visualized by a variety of tools and libraries.

After the OSM data were retreived, the process of 3D reconstruction began with a preprocessing step. First, the region containing all the buildings was identified using the maximum and minimum coordinates of the received data. The average height of the buildings was calculated at this stage as well.

**Figure 3:**
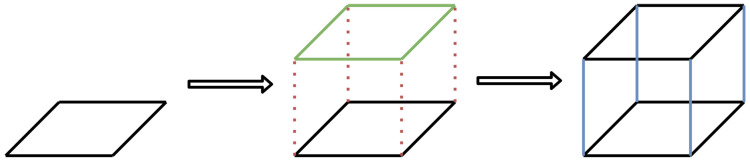
Simple building polygon reconstruction in 3D.

During the 3D reconstruction process, the polygons for each building were extracted from the OSM data. These polygons served as the foundation for the building, taking into account any gaps that may be present. If the OSM data included a height value for the building, the roof was placed at that height. Walls were then added to the building as described in Figure 3. If the OSM data included information about the type of roof, it was added on top of the building. This process was repeated for all buildings in the area.

In certain cases, a single building may be divided into multiple polygons in the OSM data in order to improve 3D visualization. However, it may be more important to prioritize data accuracy over visualization. To address this issue, the possibility of merging overlapping or adjoining buildings can be added. In our case, this was done by converting the 3D building data into a Three.js [36] mesh object and using the Constructive Solid Geometry (CSG) join operation, calling the Three-CSG [37] library. Finally, the Three.js library was used to export the 3D building data to the Wavefront OBJ or GL Transmission Format (GLTF) file format for further use. The fetched OSM area data were then parsed to OSM format using OSMtoGeoJSON [38].

The LiDAR point cloud can be used to complement the OSM data obtained through the approach described above. To process the LiDAR point cloud, we used two different tools, LasTools and PDAL. LasTools is a mostly open source command line tool for Windows platforms that can perform operations on LiDAR files, among which are decompressing files from LAZ to LAS format, generating color images from the point cloud, filtering the point cloud by LiDAR classification, etc. Several of the LasTools tools are limited in that they cannot be used on Linux, or cannot be used on Windows for LAZ files larger than 5 million points without a license.

The second tool, PDAL, is an open source command line tool for Linux and Windows platforms that can perform operations on LiDAR files. Unlike LasTools, PDAL has no file size limitations and is significantly more flexible. It is integrated in Python and allows concatenating operations on LiDAR files in pipelines in a single script. PDAL can perform operations such as filtering the point cloud by LiDAR classification, clipping the point cloud by a region of interest, reading minimum and maximum heights from the point cloud, extracting the bounding box from the point cloud, etc.

**Figure 4:**
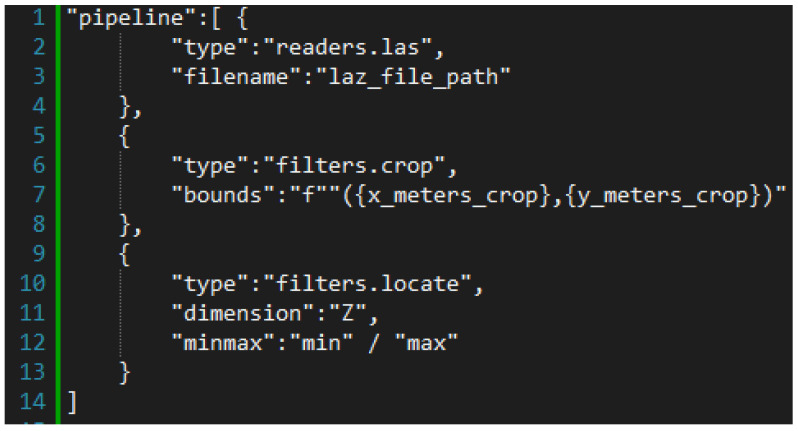
PDAL Script.

To supplement the building heights in OSM, a PDAL script was developed. The script utilizes the PDAL pipeline shown in Figure 4 for each building area.

The pipeline reads the specified LAZ file and crops it to the area of interest for the building. The minimum and maximum height for each building is then obtained. This pipeline is executed from a Python script that reads the execution result values from an array.

To remove potential noise points, filters were applied to the LAZ files to remove points that do not belong to buildings. The PDAL filter filters.range was used to remove points that do not fall within a range of values. In this case, it was used to remove points classified as vegetation (classifications 3, 4, and 5) and points classified as water (9). In addition, the PDAL filters.outlier filter was used to remove points considered as noise.

In OSM, buildings are represented by polygons with latitude and longitude. To crop an area of interest in a LAZ file with PDAL, the coordinates must be indicated as an XYZ coordinate bounding box in meters. The following steps were taken to convert the data:

1. The latitude and longitude of all vertices of the polygon representing the building are obtained.
2. The minimum and maximum latitude and longitude of the polygon vertices are obtained, generating the bounding box.
3. The information of the LAZ file is obtained using pdal info, including the projection and bounding box of the file in EPSG projection and native (in meters).
4. The range of X and Y coordinates and latitude and longitude of the LAZ file bounding box is extracted. The building bounding box is then mapped to the LAZ file bounding box (in meters).
5. The script which crops the LAZ file with PDAL and reads the minimum (ground) and maximum (roof) height of the building area is executed using the building bounding box (in meters).
6. A sensitivity parameter was added to allow increasing or decreasing the size of the bounding box of the building area, which is useful in cases where the building is not fully contained in the LAZ file or when there are buildings very close together.
7. The minimum and maximum heights obtained are stored in an array, which is then used to update the OSM data with the height values of each building.

The results of this process are discussed in Section 3.

### 2.2. Using CNN to Add Inferred LiDAR Data to OSM

An important task in the reconstruction of buildings in 3D is to correctly classify the type of roof to be added to the different buildings. This is because not all information is included when a request is made to the OSM server. In order to solve this difficulty, in this work we propose training a CNN to classify the different types of roofs given a certain geographical area, thereby completing the OSM data and allowing detailed 3D reconstruction of the buildings to be performed.

**Figure 5:**
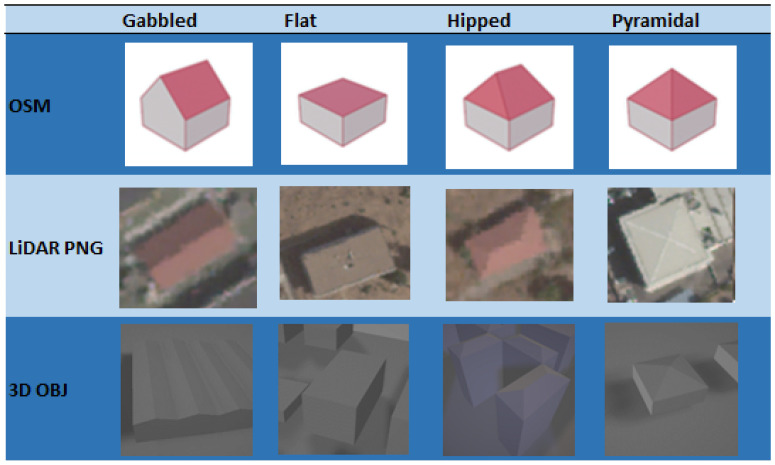
Comparison of different roof types.

In Section 1.2, we introduced CNNs as a type of neural network used in deep learning to classify images. In the particular case of this work, a CNN was implemented using the YOLOv7 repository [39], which is a version of CNN optimized for object detection in images and video in real time that is very fast, accurate, and easy to implement [40,41]. For this reason, we used CNN YOLOV7 in the experiments carried out in this work for classification of roofs for reconstructing the buildings by processing LiDAR data. Specifically, we tested whether YOLOv7 was able to recognize different roof shapes according to the roof classification provided by the OSM catalog [42]. Particularly, YOLOv7 was tested with gabled, flat, hipped, and pyramidal roofs. Figure 5 shows a comparison of the schematic view provided by OSM, the real view provided by LiDAR, and the view of the reconstructed buildings as 3D objects for each type of roof. Other roof types such as gambrel, round, and round–flat were used to train the neural network, though in smaller proportions than the former roof types.

In the next subsection, we detail the process used to detect roof shapes, starting with the preprocessing performed on the LiDAR data to build a set of images with which to train the proposed YOLOv7 CNN and then moving on to the parameters used during training.

#### 2.2.1. LiDAR Data Pre-Processing and Image Set Construction

In order to train and test the YOLOv7 CNN, we used a set of real images of rooftops. These images were taken from LiDAR, specifically a top view of six areas: Madrid, Alcalá de Henares, Córdoba, Barcelona, Zaragoza, and Luxembourg. The reason for selecting these six areas was simply that they all had a set of buildings with roofs that could be detected in order to train the neural network. In addition, all six areas have a LiDAR resolution of at least one point per square meter, which is sufficiently detailed for our purposes.

The LiDAR data were downloaded from the official website of the National Center for Geographic Information in .laz file format [43]. Processing of the information began with decompression using the laszip tool and generating a point cloud in .las format. The point cloud contained features of the entire selected geographic region, defined by classes associated with vegetation, soil, buildings, rivers, etc., as well as redundant information generated in the process of constructing the LiDAR data. For this reason, after decompression the point cloud was pre-treated with filters such as *drop_class 12*, which removes overlapping data from the point cloud, and classes 3, 4, and 5, which remove vegetation. After cleaning, surface or raster images were generated in .png format with Red, Green, and Blue (RGB) attributes and a pixel size of half a meter. For this task, the RGB and step filters of the *blast2dem* tool were used. With these generated images, the process of labeling the roofs was performed. In essence, this process consists of marking the relative position of the roofs within the geographical area, then generating coordinates within the images that provide information about the type of roof during the training process. This labeling process was performed with the Image Annotation Lab program [44]. This process, from downloading the LiDAR data to labeling the images, is summarized in the five steps shown in Table 1.

It is important to note that laszip and blast2dem are limited free software tools that come bundled with LAStools, which is a suite of tools for LiDAR data processing. While the steps summarized in Table 1 are difficult to automate, this is necessary in order to build the image set needed to train the network.

#### 2.2.2. Training the YOLOv7 CNN

Before training the network by using the set of preprocessed images to generate a model that can make inferences about roof types, it is necessary to adjust a series of parameters in order to evolve the weights used for the CNN. We used the following parameters:

1. epochs: this refers to the number of iterations performed on the image set. For training, approximately 3000 epochs were initially used, starting from a pre-trained model and performing transfer learning on it. This allowed us to create a new neural network model with updated weights based on the roof images used for training. Taking into account that the neural network incorporates features or patterns as it is trained, the updated weights model was then incorporated into the training process to continue performing transfer learning. A total of approximately 9000 additional epochs were tested, obtaining acceptable results for the final model generated using the constructed image set.
2. batchsize 2: this parameter corresponds to the number of images passed to memory per iteration within an epoch, allowing the weights of the CNN to be updated.
3. img 2000 2000: this indicates the size of the images to be resized. Because the input images all had a size of 2000 × 2000, no resizing was executed for this training.
4. weights ’model.pt’: this parameter indicates where the initial weights are located. It should be noted that the model.pt changes as training progresses over a given number of epochs, always taking the best model.

The main goal of training the YOLOv7 CNN was to generate a model able to correctly classify the roofs of buildings contained in a raster image constructed from LiDAR data. It should be noted that while the network had labels for all of the different types of roofs supported by OSM, only gabled, flat, hipped, and pyramidal roofs were supported in 3D rendering. With the correct classification of roofs, it was possible to reconstruct buildings by following the steps described below:

1. The OSM polygon representing the building is obtained.
2. The bounding box and center in terms of the latitude and longitude of the polygon are extracted.
3. The LiDAR area, transformed into an image with LASTools, is processed by the neural network. The result is a label file with the position of each detected roof in the image indicated in pixel coordinate format.
4. The labels of the image are mapped to the latitude and longitude coordinates of the building using the bounding box of the building and its center. A sensitivity parameter allows the size of the bounding box of the building to be increased or decreased, which is useful in cases where the building is not fully contained in the LiDAR area or when there are buildings very close together.
5. The roof is written to the resulting OSM file.
6. The 3D generator receives the resulting OSM and generates the 3D model.

**Figure 6:**
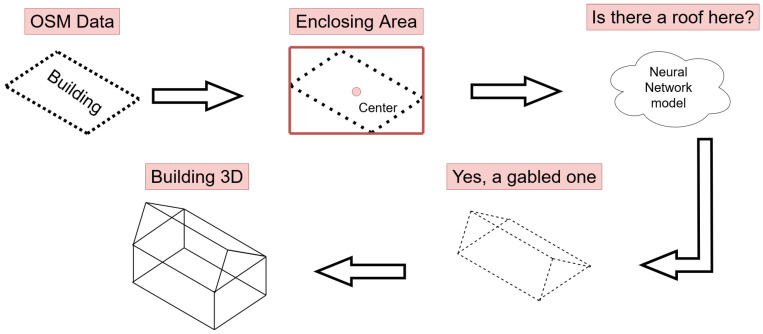
Process of adding a roof to a building using the neural network.

The steps described above and the role played by the trained YOLOv7 CNN are shown in Figure 6.

Using the network to generate this model is important, as it is later used to process the LiDAR data. This process is called post-processing, and involves using the trained model to identify a particular roof from a raster image. If the model can classify the roof, then it can provide information about its location in the image. An algorithm then uses this information to incorporate the roof into the building reconstruction.

Finally, it should be indicated that the performance evaluation of the trained network was measured using the proprietary metrics already incorporated with the YOLOv7 framework, among which we used precision, sensitivity, mAP, and F1. Precision provides the percentage of the results that are true positive (TP) from among all the positive results, which corresponds to the sum of true positives plus false positives (FP). Sensitivity, sometimes called recall, determines the proportion of the results that the trained model is able to identify as true positives from among the sum of true positives plus false negatives (FN) based on the imposed confidence. The Mean Average Precision (mAP) is a measure that combines the precision curve with the sensitivity curve, and is able to report where the developed model is both accurate and sensitive. F1 is another way of contrasting the accuracy curve against the sensitivity curve. All these metrics are based on the confusion matrix [45,46].

It is important to note that confidence is a value that reflects the extent to which the trained model finds an object within the bounding box. In other words, it is a probabilistic measure that ensures that the parameter to be estimated is within the estimated value.

In terms of execution times, the neural network was been trained for over 40 h with over 70 images and more than 1200 labels on a computer with an RTX 5000 graphic card. This is an area for future development, as better results can be achieved with more training and validation images and more time. In terms of complementing data with heights, the execution time depends on the number of buildings to be processed. The largest of the datasets we processed, Barcelona, took approximately 20 h to process on a computer with an i5 8350U and 8 GB of RAM.

## 3. Results

LiDAR files from Spain’s 2016 campaign by DGTOE of IGN were used for experimental results in Spanish cities [43], while public LiDAR data from Luxembourg Public Data were used in Luxembourg through an interactive tool [47]. The selected cities and areas were randomly chosen, with the only constraint being that the LiDAR data must have a resolution of at least one point per square meter.

Our proposed approach to supplementing building heights was tested in several areas in Spain and Luxembourg, including different areas with varying levels of building complexity; the results are shown in Table 2.

As can be seen, the amount of building data with heights varies depending on the area. This is due to the fact that OSM is a collaborative database, and not all buildings have their heights recorded. The table shows that there are more data available on buildings with heights in the city of Barcelona compared to the city of Madrid. This is because the OSM community in Barcelona has made a greater effort to add data on building heights. In the city of Madrid, the OSM community has added more data on buildings without their heights; thus, we used the proposed approach to obtain the heights of the buildings.

The number of buildings without heights is relative to the building position in the received data. In Barcelona in particular there are several buildings that collide with the limits of the LiDAR data; these have been left without heights, as the inferred heights would not have the same precision as those for the rest of the buildings. In addition, the OSM data can contain more buildings within the limits than the LiDAR data.

In less populous cities such as Córdoba, Alcalá de Henares, and Zaragoza there is a significant improvement in the amount of data on buildings that contain heights. In Luxembourg, there are almost no original heights in the OSM data on the area, resulting in a remarkable height addition percentage.

### Results Obtained Using CNN for 3D Reconstruction of Building Roofs

To obtain better post-processing performance of the generated model, and thereby guarantee better results, the CNN could have been trained with a considerable number of images; however, the neural network was trained and validated using only 40 and 39 images, respectively, which was achieved with a total of 1274 labels. In addition, it should be recalled that the selection of this set of images was made arbitrarily from LiDAR data from the town of Alcalá de Henares.

Although the set seems small, favorable practical results were obtained, mainly due to the fact that rooftops in other urban areas not found in the original training and validation image set could be inferred. Before commenting on these results, the metrics presented in the methodology and provided by YOLOv7, namely, precision, recall, mAP, and F1 [45], are discussed.

**Figure 7:**
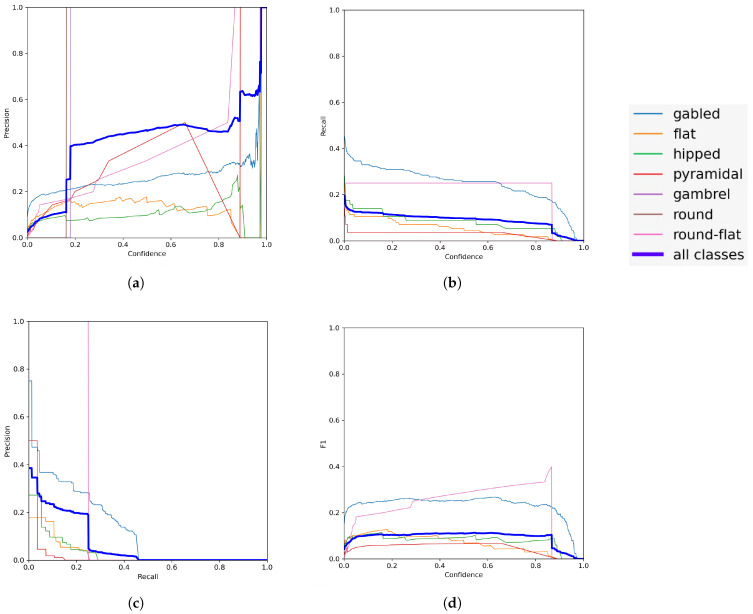
Metrics: (**a**) precision, (**b**) recall, (**c**) mAP, and (**d**) F1.

**Figure 8:**
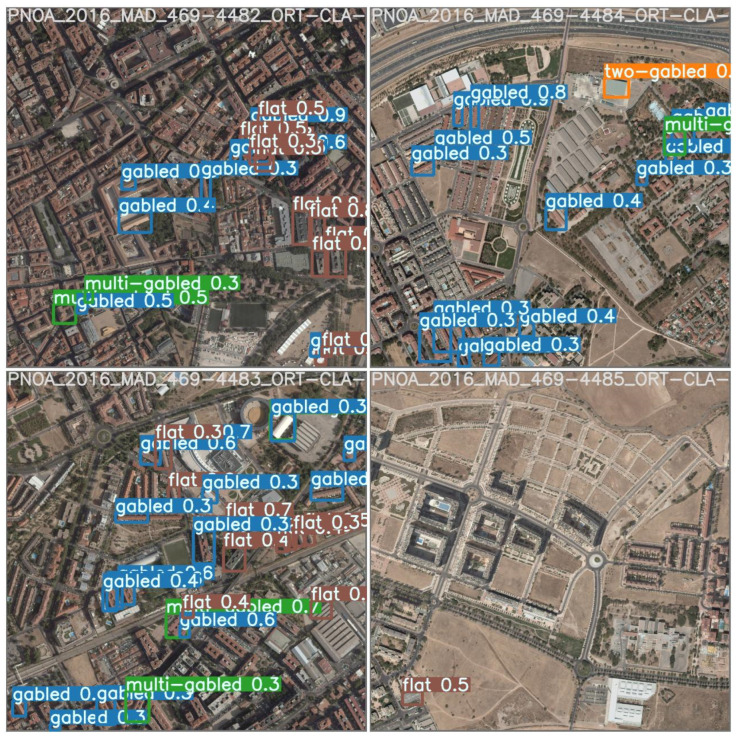
Bounding boxes of Alcalá de Henares imposed by the trained neural network model.

In Figure 7a, it can be seen that the precision is maintained between 0.4 in a confidence band that ranges from 0.2 to 0.9. In Figure 7b, it can be seen that the trained model becomes less sensitive as confidence increases. It is important to note that sensitivity is inversely proportional to accuracy, that is, the more accurate the model is, the less sensitive it will be. The mAP metric shown in Figure 7c confirms how inversely proportional precision and sensitivity are. The F1 metric shown in Figure 7d reveals the confidence value within the contrast made between precision and sensitivity, revealing an average value for both. Note that this value sits at 0.2. The difference between both mAP and F1 is that the former does not reveal the value of the trust imposed, while the latter does. Confidence is a value that reflects the extent to which the trained model finds an object within the delimiting box (see Figure 8). In Figure 7a,b,d, precision, sensitivity, and the contrast between precision and sensitivity are respectively plotted against the confidence. It can be appreciated from Figure 7a,b that as confidence increases, precision increases while sensitivity decreases. This is why the F1 curve is particularly interesting in the implementation of the trained model, as it allows the compromise between precision and sensitivity to be analyzed for a selected confidence value. Figure 7d shows a low value of precision–recall with a confidence value around 0.2. This same result can be seen in the precision–sensitivity curve shown in Figure 7c, with the difference that it does not allow the confidence value to be examined.

For the selected confidence value, Figure 7a reflects a precision around 0.4, which is quite acceptable because it means that 70% of the predictions are correct. On the other hand, for this same confidence value Figure 7b reflects a low sensitivity of less than 0.1, indicating that less than 10% of the predictions made for the same image are correct.

These results, interpreted by the metrics provided by YOLOv7, seem discouraging. However, the reality is that the neural network was trained with a very low number of images, as noted at the beginning of this section; again, it is worth noting that only 40 images were used for training and 39 for validation, with a total of 1274 labels.

Even with only the images of Alcalá de Henares used for training and validation, it was possible to identify buildings in other urban areas of Spain, namely, Cordoba, Madrid, Zaragoza, and Barcelona. Even when the whole model was tested by executing the inference on an urban area outside Spain, specifically in Luxembourg, the results were very satisfactory. In other words, by training the model with data from one area it was possible to transfer the model to other areas of the same country and even to another country. This demonstrates the robustness and generalizability of the model, as it was able to accurately classify buildings in new locations that did not contribute information to the model.

As indicated, it can be understood that these results are quite satisfactory from the point of view of the building reconstruction process. When used to process raster images generated from LiDAR, the trained neural network model reasonably classifies roofs even in urban areas not advertised in the roof classification model, allowing for roof detection and successful completion of the building reconstruction process.

To further evaluate the performance of the constructed neural network, we can examine the data in Table 3. This table presents the number of buildings in the five selected locations within Spain: Alcalá de Henares, Córdoba, Madrid, Zaragoza, and Barcelona. In addition, it presents the information for the urban area of Luxembourg, which is outside of Spain. According to the information obtained from OSM, there are 28, 288, 537, 543, 2035, and 7455 buildings in these locations, respectively (see row “N${}^{\circ }$ of buildings” in Table 3). However, OSM provides little or no information on roof type (see the “Original roofs” row in Table 3). Using our neural network trained with a limited number of samples from only the urban area of Alcalá de Henares, we can see the results obtained in the row “Added roofs”, where 17, 160, 148, 226, 392, and 2331 roofs are detected, for an average percentage of 38.82% successfully reconstructed roofs.

The results obtained with the trained neural network demonstrate that this model can be effectively used for building reconstruction. Moreover, it has been shown that satisfactory results can be obtained even with a small number of images and training epochs. Therefore, if an even larger set of images were used to train the same neural network it is likely that even better percentages of buildings with supplemented roofs could be achieved.

**Figure 9:**
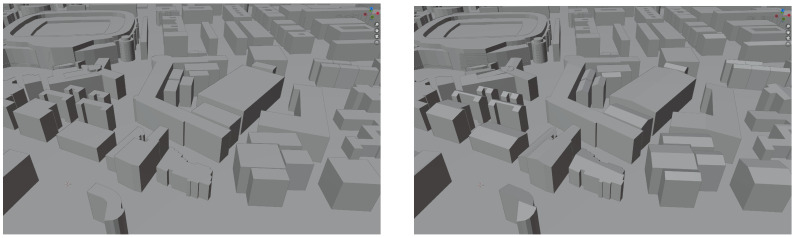
Madrid area without inferred roofs (**left**) and with inferred roofs (**right**).

Finally, in Figure 9, an urban area, close to El Paseo de la Castellana in Madrid, can be seen. In the left part of this figure, the buildings are displayed without roofs. This is because the trained neural network was not used to process this image in the reconstruction system. In the right part of the figure, the same urban area can be appreciated with added roofs. In this case, the output of the neural network has been integrated into the reconstruction system, allowing inference as to the type of roof as well as its location in the urban plan.

**Figure 10:**
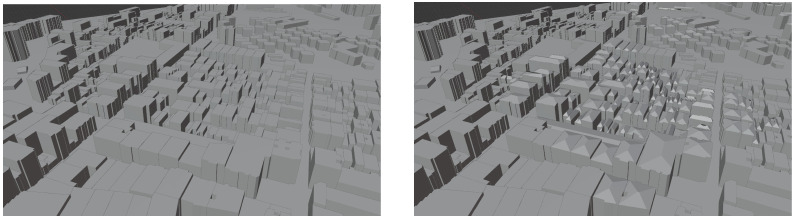
Barcelona area without inferred roofs (**left**) and with inferred roofs (**right**).

A different reconstructed area located in the city of Barcelona is shown in Figure 10. In this case, many buildings with roofs can be observed in the right part of the figure.

These results show that OSM data can be complemented with LiDAR data by processing the point cloud directly or indirectly through a neural network. The amount of data that can be complemented depends on the accuracy of the LiDAR data and the performance of the neural network. In this case, the neural network was trained with LiDAR data from the same area, resulting in relatively acceptable results. Thus, it can be verified that the neural network is able to detect buildings for which there are LiDAR data even when they are not present in OSM.

## 4. Conclusions

In this paper, a method for reconstructing 3D urban environments from OSM data supplemented with LiDAR data is proposed. A novel approach for generating 3D models of buildings and other structures from OSM data is first presented. Then, it is demonstrated how the proposed method can be complemented with different data sources, such as LiDAR data, to improve the accuracy and detail of the generated models. In particular, it is shown how LiDAR data can be processed with neural networks to extract information about the shape and slope of roofs, as well as other additional data. The results of the proposed method are compared to the original data provided by OSM.

This work has demonstrated that a better representation of the 3D environment can be obtained using LiDAR data and a CNN to improve the accuracy of OSM data.

This work does have a number of limitations. One major limitation is the cost and time required to collect and process the LiDAR data. LiDAR surveys can be expensive, particularly when covering large areas, and the data must be carefully processed and analyzed in order for it to be useful. Additionally, LiDAR data are only useful for a specific area and time period, and regular updates may be needed to maintain the accuracy of the map. This means that the results presented in the previous section could vary if the method were replicated in the future. Another challenge is the need for a large and diverse dataset of LiDAR data and corresponding building information in order to train the neural network effectively. These datasets can be difficult to obtain or generate, and may require significant pre-processing before they can be used by the neural network. This can be a time-consuming and resource-intensive task, particularly when working with large datasets.

In addition, it is important to note that not all countries have their cities mapped with LiDAR data. This means that the use of LiDAR data to improve OSM accuracy is currently only applicable to certain regions. There are several projects in Europe and North America that have mapped their cities with LiDAR; however, many other countries do not have access to this type of data. This limits the generalizability of the proposed method and its potential applications. In the Section 3, the analyzed areas are all located in Europe, where there are enough available data to test the proposed method. Furthermore, even in regions where LiDAR data are available, the coverage and resolution of the data may vary widely. This can affect the accuracy and detail of the generated 3D models, and may require the use of additional data sources or techniques to compensate for the limitations of LiDAR data. Overall, the availability and quality of LiDAR data are key factors that determine the feasibility and effectiveness of using LiDAR to improve OSM accuracy.

In future work, it would be interesting to explore different approaches for improving the performance of the neural network and for extracting more information from the LiDAR data. This could involve using more and different types of data for training the network, such as satellite images or street-level photographs, as well as using more advanced neural network architectures. Additionally, it would be useful to evaluate the performance of the method in different urban environments and with different types of OSM data and LiDAR data. Furthermore, it would be interesting to compare the results of the proposed method with other approaches for generating 3D models from OSM and LiDAR data, such as point cloud segmentation or voxel-based approaches. Another area for future research could be in the use of data augmentation techniques to increase the size and robustness of the training dataset, which can help to reduce overfitting and improve the generalization ability of the neural network.

In the future, it may be worthwhile to consider alternative approaches to reconstruct 3D buildings, such as using machine learning techniques to directly process the point cloud rather than generating images from it. Techniques such as point cloud classification or segmentation could potentially extract more detailed and accurate information from the LiDAR data, allowing for a more efficient reconstruction process. Other data that could be extracted with LiDAR include vegetation and trees, as well as roofs with shapes that are different from those used in this work.

## Figures and Tables

**Table 1:** Process for generating an image set using LIDAR data.

Process	Tool/Filters	Comments
Download	Web browser	Download LIDAR data in .laz format
Descompression	laszip	Generate point cloud in .las format
Cleanup	blast2dem/drop_class	Overlap and vegetation points are removed
Generation	blast2dem	Images are generated in png format
Labeling	Image Annotation Lab	Mark coordinates of rooftops

**Table 2:** Height results.

	Alcalá	Luxemb.	Córdoba	Madrid	Zaragoza	Barcelona
N${}^{\circ }$ of buildings	28	288	537	543	2035	7455
Original heights	1	1	27	200	17	7238
Added heights	27	287	533	336	2016	133
Buildings left without height	0	0	4	7	2	84
% of added heights	96.42%	99.65%	94.67%	61.87%	99.06%	1.78%

**Table 3:** Roof results.

	Alcalá	Luxemb.	Córdoba	Madrid	Zaragoza	Barcelona
N${}^{\circ }$ of buildings	28	288	537	543	2035	7455
Original roofs	0	0	0	4	0	174
Added roofs	17	160	148	226	392	2331
Buildings left without roof	12	128	389	334	1643	5124
% of added roofs	60.71%	55.55%	27.56%	40.88%	19.26%	28.93%

## Data Availability

Not applicable.

## Abbreviations

The following abbreviations are used in this manuscript:

OSM	OpenStreetMap
CNN	Convolutional Neural Network
DEM	Digital Elevation Model
DFM	Digital Feature Model
DTM	Digital Terrain Model
DSM	Digital Surface Model
DCNN	Dynamic Convolutional Neural Network
HTTPS	Hypertext Transfer Protocol Secure
JSON	JavaScript Object Notation
CSG	Constructive Solid Geometry
HTTP	Hypertext Transfer Protocol Secure
JSON	JavaScript Object Notation
CSG	Constructive Solid Geometry
GLTF	GL Transmission Format
DGTOE	Direction General of Teledetection and Earth Observation
IGN	National Geographic Institute
GPU	Graphics Processing Unit
RGB	Red, Green, Blue

## References

[1] Biljecki F., Stoter J., Ledoux H., Zlatanova S., Çöltekin A. (2015). Applications of 3D City Models: State of the Art Review. ISPRS Int. J. Geo-Inf..

[2] Cappelle C., El Najjar M.E., Charpillet F., Pomorski D. (2012). Virtual 3D City Model for Navigation in Urban Areas. J. Intell. Robot. Syst..

[3] Jovanović D., Milovanov S., Ruskovski I., Govedarica M., Sladić D., Radulović A., Pajić V. (2020). Building Virtual 3D City Model for Smart Cities Applications: A Case Study on Campus Area of the University of Novi Sad. ISPRS Int. J. Geo-Inf..

[4] Xu J., Liu J., Yin H., Wu T., Qiu G. Research on 3D modeling and application in urban emergency management. Proceedings of the 2011 International Conference on E-Business and E-Government (ICEE).

[5] Tayebi A., Gomez J., Saez de Adana F., Gutierrez O., Fernandez de Sevilla M. (2019). Development of a Web-Based Simulation Tool to Estimate the Path Loss in Outdoor Environments using OpenStreetMaps [Wireless Corner]. IEEE Antennas Propag. Mag..

[6] Štular B., Eichert S., Lozić E. (2021). Airborne LiDAR Point Cloud Processing for Archaeology. Pipeline and QGIS Toolbox. Remote Sens..

[7] Muhadi N.A., Abdullah A.F., Bejo S.K., Mahadi M.R., Mijic A. (2020). The use of LiDAR-derived DEM in flood applications: A review. Remote Sens..

[8] Lu J., Wang H., Qin S., Cao L., Pu R., Li G., Sun J. (2020). Estimation of aboveground biomass of Robinia pseudoacacia forest in the Yellow River Delta based on UAV and Backpack LiDAR point clouds. Int. J. Appl. Earth Obs. Geoinf..

[9] Royo S., Ballesta-Garcia M. (2019). An overview of lidar imaging systems for autonomous vehicles. Appl. Sci..

[10] Abdullah S.M., Awrangjeb M., Lu G. Automatic segmentation of LiDAR point cloud data at different height levels for 3D building extraction. Proceedings of the 2014 IEEE International Conference on Multimedia and Expo Workshops (ICMEW).

[11] Gamal A., Wibisono A., Wicaksono S.B., Abyan M.A., Hamid N., Wisesa H.A., Jatmiko W., Ardhianto R. (2020). Automatic LIDAR building segmentation based on DGCNN and euclidean clustering. J. Big Data.

[12] Garwood T.L., Hughes B.R., O’Connor D., Calautit J.K., Oates M.R., Hodgson T. (2018). A framework for producing gbXML building geometry from Point Clouds for accurate and efficient Building Energy Modelling. Appl. Energy.

[13] Yang C., Rottensteiner F., Heipke C. (2021). A hierarchical deep learning framework for the consistent classification of land use objects in geospatial databases. ISPRS J. Photogramm. Remote Sens..

[14] Pratiwi N.K.C., Fu’adah Y.N., Edwar E. (2021). Early Detection of Deforestation through Satellite Land Geospatial Images based on CNN Architecture. J. Infotel.

[15] Guo W., Yang W., Zhang H., Hua G. (2018). Geospatial object detection in high resolution satellite images based on multi-scale convolutional neural network. Remote Sens..

[16] Wang Y., Yan J., Yang Z., Jing Q., Wang J., Geng Y. (2022). GAN and CNN for imbalanced partial discharge pattern recognition in GIS. High Volt..

[17] Jadhav J., Rao Surampudi S., Alagirisamy M. (2021). Convolution neural network based infection transmission analysis on Covid-19 using GIS and Covid data materials. Mater. Today Proc..

[18] Malaainine M.E.I., Lechgar H., Rhinane H. (2021). YOLOv2 Deep Learning Model and GIS Based Algorithms for Vehicle Tracking. J. Geogr. Inf. Syst..

[19] Chun P.J., Yamane T., Tsuzuki Y. (2021). Automatic detection of cracks in asphalt pavement using deep learning to overcome weaknesses in images and gis visualization. Appl. Sci..

[20] Zhou X., Zhu M., Leonardos S., Daniilidis K. (2017). Sparse Representation for 3D Shape Estimation: A Convex Relaxation Approach. IEEE Trans. Pattern Anal. Mach. Intell..

[21] Sinha A., Bai J., Ramani K., Leibe B., Matas J., Sebe N., Welling M. (2016). Deep Learning 3D Shape Surfaces Using Geometry Images. Computer Vision—ECCV 2016, Proceedings of the European Conference on Computer Vision 2016, Amsterdam, The Netherlands, 11–14 October 2016.

[22] Neis P., Zipf A. (2012). Analyzing the Contributor Activity of a Volunteered Geographic Information Project—The Case of OpenStreetMap. ISPRS Int. J. Geo-Inf..

[23] Tyrasd (2022). Node Density Map. https://tyrasd.github.io/osm-node-density/#2/38.0/13.0/2021,places.

[24] Almendros-Jiménez J.M., Becerra-Terón A., Merayo M.G., Núñez M. (2021). Metamorphic testing of OpenStreetMap. Inf. Softw. Technol..

[25] Hagenmeyer V., KemalÇakmak H., Düpmeier C., Faulwasser T., Isele J., Keller H.B. (2016). Information and Communication Technology in Energy Lab 2.0: Smart Energies System Simulation and Control Center with an Open-Street-Map-Based Power Flow Simulation Example. Energy Technol..

[26] Ariyanto R., Syaifudin Y.W., Puspitasari D., Suprihatin, Ananta A.Y., Setiawan A., Rohadi E. (2019). A web and mobile GIS for identifying areas within the radius affected by natural disasters based on openstreetmap data. Int. J. Online Biomed. Eng..

[27] Juhász L., Novack T., Hochmair H.H., Qiao S. (2020). Cartographic Vandalism in the Era of Location-Based Games-The Case of Open Street Map and Pokémon GO. ISPRS Int. J. Geo-Inf..

[28] Fan W., Wu C., Wang J. (2019). Improving Impervious Surface Estimation by Using Remote Sensed Imagery Combined with Open Street Map Points-of-Interest (POI) Data. IEEE J. Sel. Top. Appl. Earth Obs. Remote Sens..

[29] Weiss D.J., Nelson A., Gibson H.S., Temperley W., Peedell S., Lieber A., Hancher M., Poyart E., Belchior S., Fullman N. (2018). A global map of travel time to cities to assess inequalities in accessibility in 2015. Nature.

[30] Klimanova O., Kolbowsky E., Illarionova O. (2018). Impacts of urbanization on green infrastructure ecosystem services: The case study of post-soviet Moscow. BELGEO.

[31] Bíl M., Andrášik R., Nezval V., Bílová M. (2017). Identifying locations along railway networks with the highest tree fall hazard. Appl. Geogr..

[32] Gharaee Z., Kowshik S., Stromann O., Felsberg M. (2021). Graph representation learning for road type classification. Pattern Recognit..

[33] Stewart C., Lazzarini M., Luna A., Albani S. (2020). Deep learning with open data for desert road mapping. Remote Sens..

[34] Esch T., Zeidler J., Palacios-Lopez D., Marconcini M., Roth A., Mönks M., Dech S. (2020). Towards a large-scale 3D modeling of the built environment: Joint analysis of tanDEM-X, sentinel-2 and open street map data. Remote Sens..

[35] Atwal K.S., Anderson T., Pfoser D., Züfle A. (2022). Predicting building types using OpenStreetMap. Sci. Rep..

[36] Cabello R. ThreeJS. https://threejs.org/.

[37] Alexander S. Constructive Solid Geometry for Three.js. https://github.com/samalexander/three-csg-ts.

[38] Raifer M. OSM to GeoJSON. https://github.com/tyrasd/osmtogeojson.

[39] Wang C.Y., Bochkovskiy A., Liao H.Y.M. YOLOv7 Repository. https://github.com/WongKinYiu/yolov7.

[40] Li C., Li L., Jiang H., Weng K., Geng Y., Li L., Ke Z., Li Q., Cheng M., Nie W. (2022). YOLOv6: A Single-Stage Object Detection Framework for Industrial Applications. arXiv.

[41] Redmon J., Divvala S., Girshick R., Farhadi A. You only look once: Unified, real-time object detection. Proceedings of the IEEE Conference on Computer Vision and Pattern Recognition.

[42] OpenStreetMap Key:roof:shape. https://wiki.openstreetmap.org/wiki/Key:roof:shape.

[43] Nacional I.G. Centro de Descargas del CNIG. https://centrodedescargas.cnig.es/CentroDescargas/buscador.do.

[44] 4SmartMachines Image Annotation Lab. https://ial.4smartmachines.com/.

[45] Géron A. (2022). Hands-on Machine Learning with Scikit-Learn, Keras, and TensorFlow.

[46] Skansi S. (2018). Introduction to Deep Learning: From Logical Calculus to Artificial Intelligence.

[47] Moraisferreira D. Luxembourg LiDAR Coverage Map. https://davidmoraisferreira.github.io/lidar-coverage-map-luxembourg/index.htmln.

